# Association of Epidural Analgesia in Women in Labor With Neonatal and Childhood Outcomes in a Population Cohort

**DOI:** 10.1001/jamanetworkopen.2021.31683

**Published:** 2021-10-28

**Authors:** Rachel J. Kearns, Martin Shaw, Piotr S. Gromski, Stamatina Iliodromiti, Deborah A. Lawlor, Scott M. Nelson

**Affiliations:** 1 Department of Anesthesia, Glasgow Royal Infirmary, United Kingdom; 2 School of Medicine, University of Glasgow, United Kingdom; 3 Department of Medical Physics, National Health Service Greater Glasgow and Clyde, United Kingdom; 4 Centre for Women’s Health, Institute of Population Health Sciences, Queen Mary University, London, United Kingdom; 5 Medical Research Council Integrative Epidemiology Unit at the University of Bristol, United Kingdom; 6 Population Health Science, Bristol Medical School, United Kingdom; 7 Bristol National Institute for Health Research Biomedical Research Centre, Bristol, United Kingdom

## Abstract

**Importance:**

Although use of epidural analgesia during labor is safe, detailed information about its association with neonatal and child outcomes is limited.

**Objective:**

To investigate the association of labor epidural analgesia with neonatal outcomes and childhood development during the first 1000 days of life.

**Design, Setting, and Participants:**

This population-based cohort study used Scottish National Health Service hospital administrative data of all 435 281 singleton live births in Scotland between January 1, 2007, and December 31, 2016, with follow-up over the first 1000 days of life. All 435 281 mother-infant pairs delivering between 24 weeks 0 days and 43 weeks 6 days’ gestation who were in active labor with cephalic presentation and who delivered vaginally or via unplanned cesarean delivery were included. Stillbirths and infants with known congenital anomalies were excluded. Data were analyzed between August 1, 2020, and July 23, 2021.

**Exposures:**

Epidural analgesia in labor.

**Main Outcomes and Measures:**

Neonatal outcomes included resuscitation, Apgar score less than 7 at 5 minutes, and neonatal unit admission. Childhood development measures (gross and fine motor function, communication, and social functioning) were obtained from standardized national childhood surveillance assessments performed at 2 years.

**Results:**

This study included a total of 435 281 live births with cephalic presentation in labor (median gestational age at delivery, 40 weeks [IQR, 39-41 weeks]; 221 153 male infants [50.8%]), of which 94 323 (21.7%) had labor epidural. Epidural analgesia was associated with a reduction in spontaneous vaginal deliveries (confounder-adjusted [Cadj] relative risk [RR], 0.46; 95% CI, 0.42-0.50), an increased risk of neonatal resuscitation (Cadj RR, 1.07; 95% CI, 1.03-1.11), and an increased risk of neonatal unit admission (Cadj RR, 1.14; 95% CI, 1.11-1.17). With additional analysis for mediation by mode of delivery (CMadj), these associations were reversed (CMadj RR, 0.83; 95% CI, 0.79-0.86 for neonatal resuscitation and CMadj RR, 0.94; 95% CI, 0.91-0.97 for neonatal unit admission). Epidural analgesia was associated with a reduced risk of an Apgar score less than 7 at 5 minutes in both confounder and confounder/mediation analyses. Epidural analgesia was associated with a reduced risk of having developmental concern in any domain at 2 years in confounder and confounder/mediation analyses (CMadj RR, 0.96; 95% CI, 0.93-0.98), with specifically fewer concerns regarding communication (CMadj RR, 0.96; 95% CI, 0.93-0.99) and fine motor skills (CMadj RR, 0.89; 95% CI, 0.82-0.97).

**Conclusions and Relevance:**

The results of this cohort study suggest that labor epidural analgesia is not independently associated with adverse neonatal or childhood development outcomes. Associations with neonatal resuscitation and admission were likely mediated by mode of delivery.

## Key Points

**Question:**

Is the use of epidural analgesia during labor associated with adverse neonatal and childhood outcomes?

**Findings:**

In this population-based cohort study of 435 281 mother-offspring pairs, the use of epidural analgesia in labor was not associated with adverse neonatal outcomes after adjustment for confounders and mediation by mode of delivery. Epidural analgesia was, however, associated with a small reduction in some adverse developmental outcomes at 2 years.

**Meaning:**

In this study, epidural analgesia in labor was not associated with adverse immediate or longer-term offspring outcomes.

## Teaser

This cohort study uses data on mother-infant pairs from the Scottish National Health Service to investigate the association of epidural anesthesia use during labor with neonatal and childhood outcomes.

## Introduction

Epidural analgesia is the criterion standard for pain control in labor; it is recommended by the World Health Organization and is performed in approximately 30% and 73% of laboring women in the UK and US, respectively, with rates anticipated to further increase globally.^1,2,3^ Epidurals can be medically indicated (eg, in preeclampsia) or performed at maternal request. Though highly effective, labor epidural analgesia may be associated with adverse effects including hypotension, reduced mobility, pruritus, maternal fever, fetal heart rate abnormalities, and risk of assisted vaginal or operative delivery, although the latter is contentious.^4,5^ The use of lower concentrations of local anesthetic may attenuate any increased risk of operative delivery and is therefore recommended by the American Society of Anesthesiologists/Society for Obstetric Anesthesia and Perinatology.^6^ Whether epidural analgesia in labor is associated with adverse neonatal or childhood outcomes remains incompletely understood.

Observational studies report mixed results, with some identifying an association between epidural analgesia and adverse neonatal outcomes and others not finding such a link.^7,8,9,10^ Furthermore, studies of longer-term implications of epidural use in labor on childhood developmental outcomes are scarce and not representative of modern practice.^11,12^ A 2018 Cochrane review of 40 randomized controlled trials (>11 000 women) comparing epidural use with no epidural use in labor indicated no clear difference in neonatal outcomes, and there were no data for longer-term childhood outcomes.^5^ However, the evidence was judged to be of overall low quality, limited by study inconsistency, imprecision in effect size estimates, and possible publication bias.^5^ A controversial study^13^ indicating an association between epidurals and risk of autism was criticized by 5 medical societies for significant methodological limitations, including the likelihood of residual confounding, and the findings of that study cannot be considered causal.^13,14,15^ A subsequent Canadian population-based study,^16^ which performed robust and extensive correction for confounding variables, found no association between labor epidural and autism. Statements from the American Society of Anesthesiologists and the Royal College of Anaesthetists emphasize that the analgesia currently offered to women in labor should not be altered on the basis of these findings.^14,15^

The first 1000 days of life are essential to the healthy development of the child.^17^ The American Society of Anesthesiologists has highlighted the need to foster further research on the safety of labor analgesia for mother and child.^14^ We conducted a population-based analysis of births in Scotland over a 10-year period. We hypothesized that there would be no difference in neonatal or childhood outcomes at age 2 years in children born to mothers who had received epidural analgesia in labor compared with those who had not.

## Methods

The National Health Service (NHS) Greater Glasgow and Clyde Research and Development department and the NHS Scotland Public Benefit and Privacy Panel for Health and Social Care approved all study data governance procedures for this cohort study. In accordance with NHS Scotland Public Benefit and Privacy Panel policy, participant-level consent was not required, as the electronic Data Research and Innovation Service (eDRIS) of NHS Scotland deidentified all data before analysis. Data were analyzed and reported in accordance with the Strengthening the Reporting of Observational Studies in Epidemiology (STROBE) reporting guideline.^18^

### Data Sources and Study Population

We linked 6 Scottish population databases: the Scottish Morbidity Record-02 (SMR02), the Scottish Birth Record, the National Records for Scotland, the Scottish Stillbirth Infant Death Survey, the Scottish Morbidity Record-01 (SMR01), and the Child Health Surveillance System Programme. The SMR02 records information on all women and births (approximately 54 000 per year) admitted to maternity units in the 14 Scottish NHS Boards. Data are reported at a population rather than hospital level in keeping with eDRIS policy. The Information Services Division of NHS Scotland regularly review data to assess completeness, and SMR02 data were found to be more than 90% complete at last assessment in 2017-2018.^19^ The Scottish Birth Record records neonatal care for each birth in Scotland. The National Records for Scotland registers all Scottish births, stillbirths, and infant deaths, and the Scottish Stillbirth and Infant Death Survey obtains additional information for each case. The General Registrar for Scotland collates information from death certificates. The SMR01 records all inpatient and day-case admissions to NHS hospitals and classifies diseases according to the *International Classification of Diseases, Ninth Revision* (*ICD-9*) or the *International Statistical Classification of Diseases and Related Health Problems, Tenth Revision* (*ICD-10*). The Child Health Surveillance System assesses health and development at defined time points using standardized methodology. Data quality checks are performed regularly by the Information Services Division, with 87% of records complete for all domains when last assessed.^20^

### Inclusion and Exclusion Criteria

**Figure 1.**
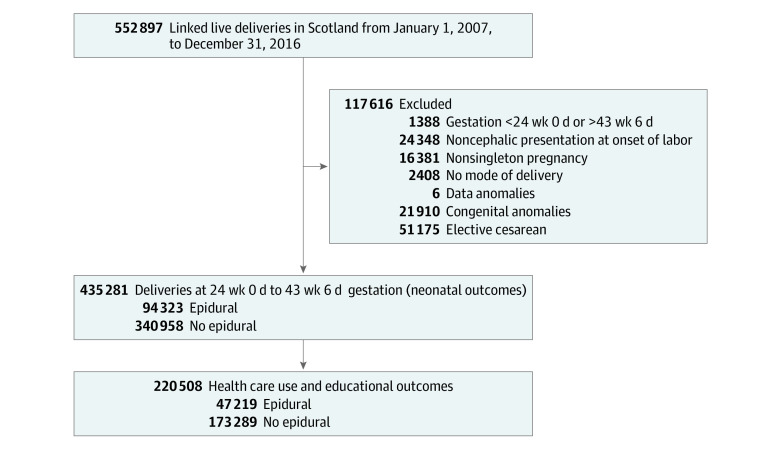
Definition of Cohort for Analysis

We linked data for all births in Scotland between January 1, 2007, and December 31, 2016, inclusive, to reflect the widespread use of low-dose epidural local anesthetic regimens. We restricted analyses to singleton live births with gestational age at delivery of 24 weeks 0 days to 43 weeks 6 days. Births were excluded if fetal presentation at the onset of labor was noncephalic, the fetus was stillborn before delivery, no mode of delivery was recorded, there was a congenital anomaly, or birth was by elective cesarean delivery (Figure 1).

### Definitions

Cephalic presentation was defined as occipitoanterior, occipitoposterior, or occipitolateral. Alternative presentations recorded as face, brow, shoulder, breech, or cord were excluded. Mode of delivery was classified as spontaneous vaginal delivery, emergency (unscheduled) cesarean delivery, instrumental (low-cavity or midcavity nonrotational forceps or nonrotational ventouse), or rotational delivery (high-cavity forceps, rotational forceps, or rotational ventouse). In SMR02, the classification *epidural* refers to lumbar epidural in labor (not combined spinal epidural, which is recorded as *spinal*). The use of combined spinal epidural for labor analgesia is unusual in Scotland and accounted for only 1% of epidurals in labor in a UK-wide survey of epidural practice.^21^

The Scottish Index for Multiple Deprivation was used to categorize residential area deprivation, with decile 1 representing the most deprived and decile 10 representing the least deprived.^22^ Data regarding delivery hospital were not available. Ethnicity, as defined by NHS Scotland 2011 census categories, was included as a covariate owing to its potential associations with both receiving epidural analgesia in labor and offspring outcomes.^23^ Smoking status at booking was labeled as current, former, or never. Gestational age at birth was defined as completed weeks of gestation based on ultrasound assessment in the first half of pregnancy. Preeclampsia was defined according to *ICD-10*. A diagnosis of diabetes (either gestational or preexisting) was recorded as diabetes or no diabetes.

Neonatal resuscitation was defined by the use of bag-mask ventilation or intubation and ventilation with or without drugs. Transient facial oxygen was classified as not having resuscitation. Apgar score at 5 minutes was reported as less than 7 or less than 4. Neonatal unit admission was dichotomized as admitted or not admitted. Data for 1- and 10-minute Apgar scores were not available. We reported offspring health care use as time spent as a hospital inpatient, number of conditions diagnosed, and number of operations performed at up to 1000 days of life. The Scottish national program of childhood health surveillance records standardized childhood development assessments on all Scottish children at defined time periods.^20^ From 2007 to 2014, assessments were performed at approximately 2 years of age and from 2012 to present, at 27 to 30 months. Between 2012 and 2014, the assessment could have been performed at either point. Health visitors assessed development in gross motor, fine motor, social, and communication domains, with results scored as either *concern* or *no concern*. Age corrected for gestation at birth was used for all assessments. From this point onward, we refer to this assessment as the 2-year child health surveillance assessment. We also report a composite outcome of any concern noted in 1 or more of the developmental assessments.

### Statistical Analysis

As this was a whole population study, no sample size calculation was performed. We used multivariable Poisson regression models with cluster robust errors (to account for more than 1 birth in some women) to determine adjusted absolute risks and relative risks (RRs) for epidural compared with any other nonneuraxial analgesic modality in labor. A robust sandwich estimator was used under the generalized estimation equation framework to correct the inflated variance found from the standard Poisson model.^24^ We used robust Poisson modeling in place of a log-binomial model to calculate RRs to avoid problems with convergence and to partially mitigate any potential estimation bias due to uncontrolled confounding. We used a multivariate Poisson regression model for childhood development assessments, taking into account the linked nature of the individual assessments (eg, a child with concern in communication may be more likely to have concern noted in social skills). Standard Poisson regression modeling was used to estimate incidence rate ratios for number of days in hospital and number of unique conditions or operations. In all analyses, *no epidural* was used as the reference category, with outcomes for *epidural* reported as RRs. Confounder-adjusted (Cadj) models were adjusted for factors that were identified a priori as having an association with neonatal and childhood outcomes but that could not be on a causal path from epidural insertion to outcome. These factors included maternal age, weight, height, ethnicity, socioeconomic deprivation, smoking, illicit drug use, induction of labor, parity, previous cesarean section, previous spontaneous or therapeutic abortion, preeclampsia, diabetes, year of birth, gestation, birthweight (as a proxy for estimated fetal weight), and fetal sex. As mode of delivery was considered to be a potential mediator in the association between epidural analgesia and neonatal and childhood development outcomes,^25^ we performed additional confounder plus mediation analyses (CMadj).

We performed several post hoc sensitivity analyses. First, we performed a propensity match to account for confounding by indication in women who received epidural analgesia compared with those who did not. Patients were matched on the covariates included in the primary analysis. We used 1:1 matching without replacement using nearest neighbor matching. Balance in maternal characteristics between groups was assessed using absolute standardized difference, with less than 0.1 considered as acceptable balance (eFigures 1 and 2 in the Supplement). Within the propensity-matched cohort, we estimated the RRs and 95% CIs for the outcomes. Second, as the timing of childhood development assessment changed over the study period, we performed a subgroup analysis of childhood development outcomes in births from January 1, 2012, to December 31, 2016, inclusive (eTable 6 in the Supplement). Third, to account for a potential interaction between preterm birth and neonatal or childhood outcomes, we used robust Poisson regression with nonlinear splines to model the associations of epidural vs no epidural with outcomes over the continuous spectrum of gestational age (eFigure 3 in the Supplement). Finally, a mediation sensitivity analysis was performed using a natural effect model, which can estimate both the direct and indirect effect paths.^26^ This analysis was implemented via a counterfactual framework estimated using an imputation strategy.^27^ This mediation methodology enabled the flexibility to use the previously described robust Poisson regression in conjunction with a categorical mediator (eTable 4 in the Supplement).

All missing data were imputed using multiple imputation via chained equations to form 10 imputed data sets using a predictive mean matching methodology.^28^ Ten iterations provided optimal data output stability, and 10 imputations were used to ensure accuracy of pooled variable effect size estimates.^28^ Missingness ranged from 0% for mode of delivery to 45.8% for ethnicity (eTable 1 in the Supplement). Missing data in confounders, in particular for ethnicity, was dealt with by using a robust imputation method using all available variables (including those not used in the current analysis), and we showed that distributions of characteristics were similar in nonimputed (eTable 1 in the Supplement) and imputed data sets (Table 1). Any outcome applying to 5 or fewer patients was recorded as less than or equal to 5 in order to prevent potential patient identification in keeping with eDRIS policy. *P* < .05 was used to indicate statistical significance, calculated using 2-sided Wilcoxon rank sum and χ^2^ tests as appropriate. Analyses were validated to ensure distributional assumptions were met and were undertaken using R, version 4.0.3 (R Foundation). Data were analyzed from January 1, 2007, to December 31, 2016.

**Table 1.** Maternal and Neonatal Characteristics of Patients After Exclusion of Data Missing for Anesthetic Type

Characteristic	No. (%)	
	No epidural (n = 340 958)	Epidural (n = 94 323)
Mother’s age, median (IQR), y	29 (24-33)	29 (24-33)
Mother’s weight, median (IQR), kg	67 (59-78)	68 (60-80)
Mother’s height, median (IQR), cm	165 (160-169)	164 (160-168)
Ethnic group		
Asian	19 098 (5.6)	5533 (5.9)
Black	6427 (1.9)	1732 (1.8)
White	310 299 (91.0)	85 606 (90.8)
Mixed	1601 (0.5)	480 (0.5)
Other^a^	3533 (1.0)	972 (1.0)
SIMD decile^b^		
1	47 539 (13.9)	12 631 (13.4)
2	42 055 (12.3)	11 213 (11.9)
3	37 969 (11.1)	10 367 (11.0)
4	35 407 (10.4)	9715 (10.3)
5	33 948 (10.0)	8712 (9.2)
6	31 676 (9.3)	8323 (8.8)
7	30 743 (9.0)	8325 (8.8)
8	29 540 (8.7)	8883 (9.4)
9	27 501 (8.1)	8205 (8.7)
10	24 580 (7.2)	7949 (8.4)
Smoker during pregnancy		
Current	69 341 (20.3)	16 756 (17.8)
Former	40 188 (11.8)	14 575 (15.5)
Never	231 429 (67.9)	62 992 (66.8)
Injected illicit drugs, yes	3477 (1.0)	749 (0.8)
Spontaneous abortion	77 858 (22.8)	19 951 (21.2)
Therapeutic abortion, yes	27 487 (8.1)	8280 (8.8)
Parity, median (IQR)	1 (0-1)	0 (0-1)
Previous cesarean birth, median (IQR)	0 (0-0)	0 (0-0)
Induction	86 692 (25.4)	42 035 (44.6)
Estimated gestation, median (IQR), wk	40 (39-40)	40 (39-41)
Birthweight, median (IQR), g	3440 (3090-3770)	3510 (3180-3840)
Male sex	172 071 (50.5)	49 082 (52.0)
Female sex	168 887 (49.5)	45 241 (48.0)
Preeclampsia	3411 (1.0)	1909 (2.0)
Diabetes	6130 (1.8)	2323 (2.5)

Abbreviation: SIMD, Scottish Index of Multiple Deprivation.

^a^ Other refers to ethnic status recorded as “unknown” in the data provided.

^b^ The degree of social deprivation was categorized using deciles according to the Scottish Index of Multiple Deprivation with deciles of 1 (most deprived) to 10 (least deprived).

## Results

Between January 1, 2007, and December 31, 2016, after exclusions, there were 435 281 live births with cephalic presentation in labor recorded in Scotland (median [IQR] gestational age at delivery, 40 weeks [39-41 weeks]; 221 153 male infants [50.8%]), of which 94 323 (21.7%) had labor epidural and 340 958 (78.3%) did not (Figure 1). Among these live births, 19 098 mother-offspring pairs were Asian (5.6%), 6427 were Black (1.9%), 310 299 were White (91.0%), 1601 were of mixed ethnicity (0.5%), and 3533 were of other ethnicity (1.0%). Mothers receiving epidural analgesia in labor were more likely to be primiparous, to be in a higher socioeconomic group, to be nonsmokers, to have undergone induction of labor, to have preeclampsia, to have diabetes, to be having a male baby, and to be having a baby with higher birthweight (Table 1). There were 303 013 spontaneous vaginal deliveries (69.6%), 70 899 emergency cesarean deliveries (16.3%), 52 799 instrumental deliveries (12.1%), 8540 rotational deliveries (2.0%), and 28 unpredicted breech extractions (<0.01%) (Table 2).

**Table 2.** Unadjusted Crude Event Rates for All Outcomes

Outcome	Events, No. (%)		
	Total	No epidural	Epidural
**Obstetric and neonatal outcomes**			
No.	435 281	340 958	94 323
Mode of delivery			
SVD	303 013 (69.6)	268 629 (78.8)	34 384 (36.5)
Breech	28^a^ (0.006)	28 (0.008)	<5 (0.004)
Emergency cesarean delivery	70 899 (16.3)	41 631 (12.2)	29 268 (31.0)
Instrumental	52 799 (12.1)	26 668 (7.8)	26 131 (27.7)
Rotational	8540 (2.0)	4002 (1.2)	4538 (4.8)
Neonatal resuscitation	33 351 (7.7)	25 429 (7.5)	7922 (8.4)
Apgar score, points			
<7 at 5 min	6250 (1.4)	5008 (1.5)	1242 (1.3)
<4 at 5 min	1561 (0.4)	1237 (0.4)	324 (0.3)
Admitted to neonatal unit	30 211 (6.9)	23 645 (6.9)	6566 (7.0)
**Health care use and educational outcomes**			
No.	220 508	173 289	47 219
Time in hospital up to age 2 y, median (IQR), d^b^	NA	0 (0-0)	0 (0-0)
No. of unique conditions diagnosed up to age 2 y, median (IQR)	NA	0 (0-1)	0 (0-1)
No. of operations performed up to age 2 y, median (IQR)	NA	0 (0-0)	0 (0-0)
Concern at age 2 y			
Gross motor	4494 (2.0)	3657 (2.1)	837 (1.8)
Fine motor	5414 (2.5)	4446 (2.6)	968 (2.1)
Communication	29 118 (13.2)	23 336 (13.5)	5782 (12.2)
Social	9599 (4.4)	7681 (4.4)	1918 (4.1)
Any concern noted in ≥1 developmental domain	33 638 (15.3)	26 948 (15.6)	6690 (14.2)

Abbreviations: NA, not available; SVD, spontaneous vaginal delivery.

^a^ The total number of breech deliveries is 28 plus up to 5. Any outcome applying to 5 or fewer patients was recorded as ≤5 in order to prevent potential patient identification in keeping with the electronic Data Research and Innovation Service policy.

^b^ Days in hospital are counts of full days. Hospital stays are counted as 0 if <24 hours’ duration.

Epidural use was associated with a reduced chance of having a spontaneous vaginal delivery (34 384 of 94 323 cases [36.5%]) compared with no epidural (268 629 of 340 958 cases [78.8%]) (Cadj RR, 0.46; 95% CI, 0.42-0.50) (Table 2 and Table 3). On initial analyses accounting for a range of confounders, epidural use was associated with increased risk of neonatal resuscitation (Cadj RR, 1.07; 95% CI, 1.03-1.11) and neonatal unit admission (Cadj RR, 1.14; 95% CI, 1.11-1.17) (Table 3). On further sensitivity analyses investigating mediation by mode of delivery (CMadj), a competitive mediation with attenuation of the risk was present for neonatal resuscitation (CMadj RR, 0.83; 95% CI, 0.79-0.86) and neonatal unit admission (CMadj RR, 0.94; 95% CI, 0.91-0.97) (Table 3; eTable 4 in the Supplement). To account for confounding by indication, a propensity score–matched sensitivity analysis was performed that found no difference between groups for neonatal resuscitation (RR, 0.99; 95% CI, 0.95-1.04) but an increased risk of neonatal unit admission with epidural use (RR, 1.10; 95% CI, 1.06-1.14). Epidural use was associated with a decreased risk of an Apgar score less than 7 at 5 minutes in Cadj analysis (Cadj RR, 0.92; 95% CI, 0.86-0.99), mediation analysis (CMadj RR, 0.74; 95% CI, 0.69-0.79), and in propensity score–matched analysis (Cadj RR, 0.84; 95% CI, 0.78-0.91), with similar findings for the more severe outcome of an Apgar score less than 4 at 5 minutes (Table 3).

**Table 3.** Unadjusted, Confounder-Adjusted, Confounder/Mediator-Adjusted, and Propensity-Matched Relative Risks (RRs) and 95% CIs for All Outcomes Referent to Receiving No Epidural (RR = 1)^a^

Outcome	Unadjusted		Confounder adjusted (Cadj)		Confounder and mediator (mode of delivery) adjusted (CMadj)		Propensity matching (Cadj)	
	RR (95% CI)	*P* value	RR (95% CI)	*P* value	RR (95% CI)	*P* value	RR (95% CI)	*P* value
Mode of delivery								
SVD	0.46 (0.46-0.47)	<.001	0.46 (0.42-0.50)	<.001	NA	NA	NA	NA
Breech	0.26 (0.06-1.09)	.06	0.61 (0.09-4.01)	.70	NA	NA	NA	NA
Emergency cesarean delivery	2.55 (2.50-2.60)	<.001	1.62 (1.46-1.81)	<.001	NA	NA	NA	NA
Instrumental	3.54 (3.48-3.60)	<.001	2.50 (2.29-2.73)	<.001	NA	NA	NA	NA
Rotational	4.09 (3.92-4.27)	<.001	2.86 (2.15-3.81)	<.001	NA	NA	NA	NA
Neonatal resuscitation	1.15 (1.11-1.19)	<.001	1.07 (1.03-1.11)	.003	0.83 (0.79-0.86)	<.001	0.99 (0.95-1.04)	.76
Apgar score, points								
<7 at 5 min	0.91 (0.85-0.97)	.003	0.92 (0.86-0.99)	.02	0.74 (0.69-0.79)	<.001	0.84 (0.78-0.91)	<.001
<4 at 5 min	0.97 (0.85-1.10)	.60	1.04 (0.91-1.18)	.58	0.83 (0.72-0.95)	.008	1.01 (0.83-1.22)	.95
Admitted to neonatal unit	1.01 (0.98-1.03)	.70	1.14 (1.11-1.17)	<.001	0.94 (0.91-0.97)	<.001	1.10 (1.06-1.14)	<.001
Offspring								
No. of unique conditions up to age 2 y	1.03 (1.01-1.05)	<.001	1.06 (1.04-1.09)	<.001	1.06 (1.04-1.08)	<.001	1.07 (1.04-1.10)	<.001
No. of days in hospital up to age 2 y	0.92 (0.86-0.99)	.02	1.04 (0.98-1.11)	.19	1.03 (0.96-1.10)	.39	1.06 (0.97-1.15)	.20
No. of operations up to age 2 y	0.94 (0.90-0.98)	.006	1.01 (0.96-1.06)	.72	1.00 (0.95-1.05)	.86	1.01 (0.95-1.07)	.81
Concern at age 2 y								
Gross motor	0.85 (0.79-0.92)	<.001	0.95 (0.88-1.03)	.25	0.91 (0.83-0.99)	.03	0.95 (0.85-1.05)	.32
Fine motor	0.81 (0.75-0.87)	<.001	0.91 (0.84-0.98)	.01	0.89 (0.82-0.97)	.005	0.91 (0.82-1.00)	.06
Communication	0.91 (0.88-0.94)	<.001	0.96 (0.93-0.99)	.01	0.96 (0.93-0.99)	.02	0.97 (0.93-1.01)	.09
Social	0.93 (0.88-0.98)	.007	0.98 (0.93-1.04)	.52	0.95 (0.90-1.01)	.12	0.98 (0.91-1.05)	.51
Any	0.91 (0.89-0.94)	<.001	0.96 (0.93-0.98)	.001	0.96 (0.93-0.98)	<.001	0.96 (0.92-0.99)	.03

Abbreviations: Cadj, confounder adjusted; CMadj, confounder and mediator adjusted; NA, not applicable; RR, relative risk; SVD, spontaneous vaginal delivery.

^a^ Results are adjusted for maternal age, maternal weight, Scottish Index of Multiple Deprivation decile, race and ethnicity, smoking history, illicit drug use, induction of labor, parity, previous cesarean section, previous spontaneous or therapeutic abortion, preeclampsia, diabetes, gestational age, birthweight, year of birth, and sex of neonate.

**Figure 2.**
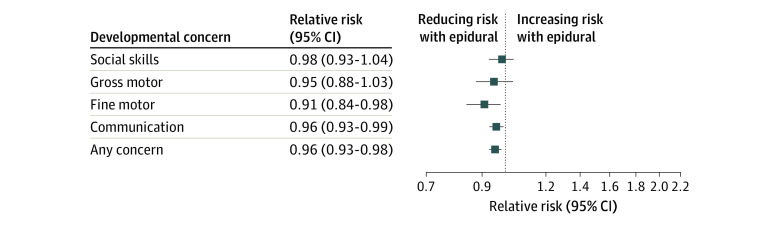
Confounder-Adjusted Relative Risks for Childhood Development Outcomes Referent to Receiving No Epidural

With respect to longer-term follow-up, we completed analyses on 220 508 mother-infant pairs, of whom 47 219 (21.4%) received an epidural during labor and 173 289 (78.6%) did not. Patients receiving follow-up were more likely to be of White ethnicity, to be from a lower socioeconomic group, to be smokers, to have used illicit drugs, and to have a male infant (eTable 5 in the Supplement). After adjustment for confounders, we observed an association between maternal epidural and number of diagnosed health conditions in the child (Cadj RR, 1.06; 95% CI, 1.04-1.09) but not with number of days in the hospital (Cadj RR, 1.04; 95% CI, 0.98-1.11) or number of operations within the first 2 years of life (Cadj RR, 1.01; 95% CI, 0.96-1.06) (Table 3). These results were unchanged when we performed additional analyses for mediation by mode of delivery and in propensity score–matched analyses. For the childhood developmental outcomes, children of mothers who had an epidural were less likely to have a concern raised in any of the developmental domains in both Cadj and CMadj analyses (CMadj RR, 0.96; 95% CI, 0.93-0.98), with specifically fewer concerns regarding their communication (CMadj RR, 0.96; 95% CI, 0.93-0.99) and fine motor skills (CMadj RR, 0.89; 95% CI, 0.82-0.97) (Figure 2 and Table 3). Effect size estimates were similar but with wider CIs when analyses were restricted to mothers whose children were born between January 1, 2012, and December 31, 2016 (eTable 6 in the Supplement). There were no differences between groups for developmental outcomes in the propensity score–matched analyses (Table 3). There was no difference between groups for any of the outcomes when analyzed over the spectrum of gestational ages (eFigure 3 in the Supplement). Results were similar across complete-case and imputed data sets and across unadjusted, adjusted, and propensity score–matched analyses (Tables 2 and 3; eTables 2-4 in the Supplement).

## Discussion

In this cohort study of 435 281 mother-infant pairs, maternal epidural analgesia in labor was associated with neonatal resuscitation, neonatal unit admission, and increased number of health conditions, but not with an Apgar score less than 7 or less than 4 at 5 minutes or with health care use or adverse childhood developmental outcomes at 2 years. Additional mediation analysis for mode of delivery reversed the associations between epidural and neonatal resuscitation and neonatal unit admission, which suggests that mode of delivery is likely a powerful mediating factor in this association. Sensitivity analyses including a propensity score–matched cohort and restriction to later time periods had similar effect size estimates. In sensitivity analyses across the range of gestational age, no differences were seen between epidural and nonepidural groups. Children of mothers who received an epidural were less likely to be identified as raising concerns about communication, fine motor function, or the composite outcome of having an abnormality in any 1 of the developmental domains in both Cadj and CMadj analyses. These findings may provide reassurance to parents and health care professionals regarding the safety of epidural analgesia.

Data from meta-analyses of randomized studies suggest no difference in neonatal morbidity as assessed by neonatal unit admission or Apgar score in mothers receiving epidural analgesia in labor.^5^ These studies were largely performed at a time dominated by different anesthetic, obstetric, and neonatal practice in highly select populations, and they predominantly compare epidural use with systemic opioid analgesia, which is known to have profound effects on fetal and neonatal responsiveness.^5^ Neonatal outcomes were not reported in trials comparing epidural with no analgesia.^5^ Trials comparing high- and low-dose epidural regimens^29^ or the addition of opioids to the epidural infusion^30^ found no differences in neonatal outcomes, but these trials are not comparable to our study comparing any epidural analgesia to no epidural. None of these meta-analyses reported longer-term childhood outcomes.^5,29,30^

Our study contrasts with some larger observational studies, including a large Swedish population-based study^8^ of 294 329 nulliparous women from 1999 to 2008, which showed an association between epidural use and low Apgar score. In that study, women who received epidural analgesia had a higher incidence of uterine dystocia, prolonged labor, and instrumental delivery resulting in concerns about confounding by indication.^8^ This specific issue was addressed in a propensity matching study of 257 872 singleton births in the Netherlands, and the association of epidural analgesia with a low Apgar score was still evident, although the authors acknowledged their inability to account for smoking status and weight.^9^ A US observational study^31^ including 106 845 operative vaginal births (gestational age between 36weeks 0 days and 41 weeks 6 days) in which 86.6% of patients received epidural analgesia found no association between epidural and neonatal morbidity. Other smaller studies are less consistent, with a retrospective review of 48 352 patients from a single center between 2008 and 2017 demonstrating an increase in operative births but no association with adverse neonatal outcomes in women who had labor epidurals.^10^ Others (n = 25 643) did not observe an increased rate of instrumental delivery but reported an increase in low Apgar score and neonatal admission with epidural use.^7^ Our study addresses many of the limitations seen in these studies by examining a whole population cohort of 435 281 mother-infant pairs from all obstetric units in Scotland over a recent 10-year period, precluding the effects of selection bias, and performing extensive and robust adjustment for potential confounding variables. We performed a range of sensitivity analyses, including a propensity score match, and found no difference in our results.

In contrast with meta-analyses of randomized clinical trials^5,29,32^ and larger observational studies,^33,34^ we observed an association between epidural and increased risk of operative delivery, despite the transition to lower doses of local anesthetic for epidurals over the last 2 decades. Women who request epidural analgesia may inherently differ from those who do not, with a higher baseline risk of requiring assisted or operative delivery. The decision to use epidural analgesia is rarely made at random, and it is more likely to be used when labor is particularly long or painful, particularly when there is a higher likelihood of requiring operative intervention.^35^ Similarly, the decision to perform an operative delivery may be influenced by a wide variety of factors, beyond those relating to the patient.^36,37^

Studies of associations between labor epidurals and longer-term childhood outcomes are scarce. A study of 4684 children born vaginally between 1976 and 1982 indicated that maternal epidural use was not associated with an increased risk of learning difficulties by age 19 years.^12^ More recently, a retrospective cohort study of 147 895 children showed a 37% relative increase in the risk of autism in babies whose mothers had epidural analgesia in labor.^13^ This report stimulated debate and statements from professional societies regarding the likelihood of residual confounding and the lack of causal analysis.^14,15^ A subsequent Canadian population-based study of 123 175 offspring found no association between labor epidural and autism, although the study did not examine other more general markers of childhood development.^16^ In our study, we focused on a routine, standardized national program of childhood surveillance assessments in 4 developmental domains, providing an overview of childhood developmental attainment at 2 years of age, and found no detrimental association with epidural use. However, we acknowledge that the subjective nature of the childhood development assessments, the difficulties inherent in assessing young children particularly in speech and language, and the large size of the data set may have contributed to the strength of the associations observed.

### Strengths and Limitations

Our study had a number of strengths, including an unselected population cohort of linked mother-infant data, robust processes for imputation of missing data, extensive adjustment for a wide range of confounding factors known to be associated with epidural analgesia, comprehensive sensitivity analyses, and prolonged follow-up over the first 2 years of life. To our knowledge, this is the largest study examining associations of epidural analgesia with neonatal and longer-term childhood development outcomes.

We acknowledge a number of limitations, including that of confounding by indication. We performed a wide range of sensitivity analyses to address this limitation, including time-limited analyses, nonlinear continuous adjustment for gestational age, propensity score–matched analysis, and mediation. Analyses of potential mediation by mode of delivery assume that there are no unadjusted confounders between mode of delivery and outcomes. Although we cannot exclude this possibility, we have adjusted for key confounders of this association, including parity, pregnancy complications, and past obstetric history. That our results were similar for both adjusted and unadjusted analyses in both nonimputed and imputed data and in propensity-matched analyses would all support our observed associations. We did not have information regarding indication for epidural, epidural drugs used, systemic opioids, use of nitrous oxide, duration of labor, dystocia, chorioamnionitis, maternal and fetal hemodynamics, or cord blood-gas status. The use of systemic opioids or nitrous oxide could have occurred in patients in both groups, but we were unable to elucidate this further. We were unable to perform analyses including the delivery hospital, as these data were not available. We accept that higher-volume centers were more likely to offer onsite anesthetic support, including epidural provision, and were more likely to house higher-risk deliveries; this likelihood could have biased our results against the epidural group. We had a reduced number of cases for childhood development outcomes. Women whose children received follow-up were more likely to be White, of a lower socioeconomic group, and smokers and to have used illicit drugs. However, our 14.2% rate of having 1 or more concerns in a developmental domain in offspring of mothers who had an epidural and 15.6% in offspring of mothers who did not have an epidural are in keeping with reported national figures of approximately 15%.^20^ Future studies with prolonged follow-up including educational outcomes would be useful and may provide further reassurance to parents that epidural analgesia is not associated with childhood developmental concerns.

## Conclusions

In conclusion, our data from a population of 435 281 mother-infant pairs in Scotland suggest that use of epidural analgesia in labor is not independently associated with adverse neonatal outcomes or adverse childhood developmental outcomes. This information may be used to aid decision-making for women considering epidural analgesia in labor.

## Notes

Supplement. **eTable 1.** Nonimputed Data: Maternal and Neonatal Characteristics of Patients (After Exclusion of Data Missing for Anesthetic Type) **eTable 2.** Nonimputed Data: Event Rates for all Outcomes **eTable 3.** Nonimputed Data: Unadjusted, Confounder-Adjusted and Confounder/Mediator Adjusted Relative Risks and 95% CI for all Outcomes for Outcomes Referent to Receiving no Epidural (RR = 1) **eTable 4.** Estimation of Direct and Indirect Effects of Epidural Mediated by Mode of Delivery on Outcomes Referent to Receiving no Epidural (RR = 1) **eTable 5.** Imputed Data: Characteristics of Women With and Without Educational Outcomes (After Exclusion of Data Missing for Anesthetic Type) **eTable 6.** Association of Epidural on Childhood Outcomes for Children Born Between January 1, 2012, December 31, 2016, Inclusive Referent to Receiving no Epidural (RR = 1) **eFigure 1.** Love Plot Illustrating the Balance in Covariates Between Unmatched (red) and Matched (blue) Data Sets **eFigure 2.** Love Plot Illustrating the Balance in Covariates Between Unmatched (red) and Matched (blue) for the Health Care Use and Educational Outcomes Data Sets **eFigure 3.** Time-Varying Absolute Risks for Each Outcome in Relation to Gestational Age in Weeks
