# Supplementary material for: Association of Epidural Analgesia in Women in Labor With Neonatal and Childhood Outcomes in a Population Cohort
Source: JAMA Netw Open. 2021 Oct 28;4(10):e2131683. doi: 10.1001/jamanetworkopen.2021.31683 (PMC8554639; doi:10.1001/jamanetworkopen.2021.31683)
Supplement: Supplement. — eTable 1. Nonimputed Data: Maternal and Neonatal Characteristics of Patients (After Exclusion of Data Missing for Anesthetic Type) eTable 2. Nonimputed Data: Event Rates for all Outcomes eTable 3. Nonimputed Data: Unadjusted, Confounder-Adjusted and Confounder/Mediator Adjusted Relative Risks and 95% CI for all Outcomes for Outcomes Referent to Receiving no Epidural (RR = 1) eTable 4. Estimation of Direct and Indirect Effects of Epidural Mediated by Mode of Delivery on Outcomes Referent to Receiving no Epidural (RR = 1) eTable 5. Imputed Data: Characteristics of Women With and Without Educational Outcomes (After Exclusion of Data Missing for Anesthetic Type) eTable 6. Association of Epidural on Childhood Outcomes for Children Born Between January 1, 2012, December 31, 2016, Inclusive Referent to Receiving no Epidural (RR = 1) eFigure 1. Love Plot Illustrating the Balance in Covariates Between Unmatched (red) and Matched (blue) Data Sets eFigure 2. Love Plot Illustrating the Balance in Covariates Between Unmatched (red) and Matched (blue) for the Health Care Use and Educational Outcomes Data Sets eFigure 3. Time-Varying Absolute Risks for Each Outcome in Relation to Gestational Age in Weeks [file jamanetwopen-e2131683-s001.pdf]

# Supplementary Online Content

Kearns RJ, Shaw M, Gromski PS, Iliodromiti S, Lawlor DA, Nelson SM. Association of epidural analgesia in women in labor with neonatal and childhood outcomes in a population cohort. *JAMA Netw Open*. 2021;4(10):e2131683. doi:10.1001/jamanetworkopen.2021.31683

**eTable 1.** Nonimputed Data: Maternal and Neonatal Characteristics of Patients (After Exclusion of Data Missing for Anesthetic Type)

**eTable 2.** Nonimputed Data: Event Rates for all Outcomes

**eTable 3.** Nonimputed Data: Unadjusted, Confounder-Adjusted and Confounder/Mediator Adjusted Relative Risks and 95% CI for all Outcomes for Outcomes Referent to Receiving no Epidural (RR = 1)

**eTable 4.** Estimation of Direct and Indirect Effects of Epidural Mediated by Mode of Delivery on Outcomes Referent to Receiving no Epidural (RR = 1)

**eTable 5.** Imputed Data: Characteristics of Women With and Without Educational Outcomes (After Exclusion of Data Missing for Anesthetic Type)

**eTable 6.** Association of Epidural on Childhood Outcomes for Children Born Between January 1, 2012, December 31, 2016, Inclusive Referent to Receiving no Epidural (RR = 1)

**eFigure 1.** Love Plot Illustrating the Balance in Covariates Between Unmatched (red) and Matched (blue) Data Sets

**eFigure 2.** Love Plot Illustrating the Balance in Covariates Between Unmatched (red) and Matched (blue) for the Health Care Use and Educational Outcomes Data Sets

**eFigure 3.** Time-Varying Absolute Risks for Each Outcome in Relation to Gestational Age in Weeks

This supplementary material has been provided by the authors to give readers additional information about their work.

© 2021 Kearns RJ et al. *JAMA Network Open.*

**eTable 1.** Nonimputed Data: Maternal and Neonatal Characteristics of Patients (After Exclusion of Data Missing for Anesthetic Type)

| Total (n=427,266) | | | | |
|---|---|---|---|---|
| | | **Missing data** | **No epidural (n=335,598)** | **Epidural (n=91,668)** |
| Age of mother | | 0 | 29 (24–33) | 29 (24–33) |
| Weight of mother | | 58,458 (13.4%) | 67 (59–78) | 68 (60–80) |
| Height of mother | | 59,489 (13.7%) | 164 (160–169) | 164 (160–168) |
| Ethnic group | Asian | 199,369 (45.8%) | 7,887 (4.4%) | 2,579 (4.6%) |
| | Black | | 2,907 (1.6%) | 815 (1.5%) |
| | Mixed | | 755 (0.4%) | 269 (0.5%) |
| | Other | | 256 (0.9%) | 477 (0.9%) |
| | White | | 164,886 (92.6%) | 51,348 (92.5%) |
| SIMD decile[a] | 1 | 1,056 (0.24%) | 46,488 (13.9%) | 12,074 (13.2%) |
| | 2 | | 41,394 (12.4%) | 10,918 (11.9%) |
| | 3 | | 37,345 (11.2%) | 10,095 (11.0%) |
| | 4 | | 34,814 (10.4%) | 9,453 (10.3%) |
| | 5 | | 33,329 (10.0%) | 8,465 (9.3%) |
| | 6 | | 31,102 (9.3%) | 8,048 (8.8%) |
| | 7 | | 30,154 (9.0%) | 8,068 (8.8%) |
| | 8 | | 28,991 (8.7%) | 8,590 (9.4%) |
| | 9 | | 26,981 (8.1%) | 7,939 (8.7%) |
| | 10 | | 24,235 (7.2%) | 7,750 (8.5%) |
| Smoker during pregnancy | Current | 27,529 (6.3%) | 64,160 (20.4%) | 15,444 (17.7%) |
| | Former | | 37,144 (11.8%) | 13,606 (15.6%) |
| | Never | | 213,679 (67.8%) | 58,067 (66.7%) |
| Injected illicit drugs – YES | | 154,745 (35.6%) | 1,906 (0.9%) | 481 (0.8%) |
| Spontaneous abortion - YES | | 1,180 (0.3%) | 76,439 (22.8%) | 19,278 (21.1%) |
| Therapeutic abortion - YES | | 1,177 (0.3%) | 27,124 (8.1%) | 8,081 (8.8%) |
| Parity | | 1,690 (0.4%) | 1 (0–1) | 0 (0-1) |
| Previous CS | | 1,519 (0.3%) | 0 (0–0) | 0 (0–0) |
| Induction | | 2,506 (0.6%) | 84,747 (25.3%) | 40,602 (44.3%) |
| Est gestation | | 0 | 40 (39–40) | 40 (39–41) |
| Birthweight | | 460 (0.1%) | 3,440 (3,092–3,770) | 3,510 (3,180–3,840) |
| Male sex | | 16 (<0.01%) | 169,287 (50.4%) | 47,628 (52.0%) |
| Pre–eclampsia | | 0 | 3,310 (1%) | 1,839 (2%) |
| Diabetes | | 28,375 (6.5%) | 5,672 (1.8%) | 2,208 (2.5%) |

Data are no. (%) or median (IQR).

[a]The degree of social deprivation was categorised using deciles according to the Scottish Index of Multiple Deprivation (SIMD) with deciles of 1 (most deprived) to 10 (least deprived).

© 2021 Kearns RJ et al. *JAMA Network Open.*

**eTable 2.** Nonimputed Data: Event Rates for all Outcomes

| Neonatal outcomes | | | |
|---|---|---|---|
| | **Total no. of events** | **No epidural** | **Epidural** |
| SVD | 299,689/427,266 (68.8%) | 265,612 (79.1%) | 34,077 (37.2%) |
| Breech | 29/427,266 (0.01%) | 27 (<0.01%) | 2 (<0.01%) |
| Emergency CS | 67,064/427,266 (15.5%) | 39,699 (11.8%) | 27,365 (29.9%) |
| Instrumental | 52,012/427,266 (11.9%) | 26,285 (7.8%) | 25,727 (28.1%) |
| Rotational | 84,72/427,266 (1.9%) | 3,975 (1.2%) | 4,497 (4.9%) |
| Neonatal resuscitation | 26,763/393,694 (6.7%) | 20,398 (6.6%) | 6,365 (7.5%) |
| Apgar score < 7 at 5-mins | 6,037/423,406 (1.4%) | 4,841 (1.5%) | 1,196 (1.3%) |
| Admitted to neonatal unit | 28,896/423,017 (6.7%) | 22,667 (6.8%) | 6,229 (6.8%) |
| **Healthcare utilization and educational outcomes** | | | |
| | **Total no. of events** | **No epidural** | **Epidural** |
| Days in hospital [a,b] | ‥ | 0 (0–0) | 0 (0–0) |
| No. of unique conditions [a] | ‥ | 0 (0–1) | 0 (0–1) |
| No. of operations | .. | 0 (0-0) | 0 (0-0) |
| Gross motor concern | 4,029/203,591 (2.0%) | 3,278 (2.0%) | 751 (1.7%) |
| Fine motor concern | 4,673/202,423 (2.3%) | 3,836 (2.4%) | 837 (1.9%) |
| Communication concern | 26,825/205,142 (13.1%) | 21,530 (13.3%) | 5,295 (12.1%) |
| Social concern | 8,734/205,416 (4.3%) | 6,980 (4.3%) | 1,754 (4.0%) |
| Any concern noted | 28,839/198,570 (14.5%) | 23,175 (14.8%) | 5,664 (13.4%) |

[a]Data are N (%) or median (range). [b]Days in hospital are counts of full days. Hospital stays are counted as 0 if less than 24 hours duration.

© 2021 Kearns RJ et al. *JAMA Network Open.*

**eTable 3.** Nonimputed Data: Unadjusted, Confounder-Adjusted and Confounder/Mediator Adjusted Relative Risks and 95% CI for all Outcomes for Outcomes Referent to Receiving no Epidural (RR = 1)

| | **Epidural (unadjusted)** | | **Epidural (confounder adjusted) [Cadj]** | | **Epidural (confounder and mediator [mode of delivery] adjusted) [CMadj]** | |
|---|---|---|---|---|---|---|
| | **RR (95% CI)** | ***P* value** | **RR (95% CI)** | ***P* value** | **RR (95% CI)** | ***P* value** |
| SVD | 0.47 (0.47-0.47) | <.001 | 0.51 (0.51-0.52) | <.001 | - | - |
| Breech | 0.27 (0.05-1.47) | .11 | 0.41 (0.41-0.42) | <.001 | - | - |
| Emergency CS | 2.52 (2.48-2.56) | <.001 | 1.66 (1.62-1.69) | <.001 | - | - |
| Instrumental | 3.58 (3.52-3.65) | <.001 | 3.23 (3.18-3.29) | <.001 | - | - |
| Rotational | 4.14 (3.94-4.35) | <.001 | 2.49 (2.48-2.51) | <.001 | - | - |
| Neonatal resuscitation | 1.14 (1.11-1.17) | <.001 | 1.02 (0.98-1.06) | .36 | 0.80 (0.76-0.85) | <.001 |
| Apgar < 7 at 5 minutes | 0.90 (0.84-0.96) | .001 | 0.81 (0.70-0.91) | <.001 | 0.68 (0.58-0.78) | <.001 |
| Admitted to neonatal unit | 1.00 (0.98-1.03) | .82 | 1.09 (1.05-1.13) | <.001 | 0.92 (0.88-0.96) | <.001 |
| **Healthcare utilization and educational outcomes** | | | | | | |
| No. of unique conditions | 1.03 (1.01-1.05) | <.001 | 1.04 (1.01-1.07) | .004 | 1.04 (1.02-1.07) | .003 |
| No. of days in hospital | 0.92 (0.86-0.99) | .02 | 1.06 (0.98-1.14) | .17 | 1.06 (0.98-1.14) | .15 |
| No. of operations | 0.94 (0.89-0.98) | .005 | 1.00 (0.93-1.07) | .94 | 0.98 (0.91-1.05) | .63 |
| Gross motor concern | 0.86 (0.77-0.94) | <.001 | 0.97 (0.85-1.08) | .56 | 0.91 (0.79-1.03) | .14 |
| Fine motor concern | 0.81 (0.73-0.89) | <.001 | 0.90 (0.79-1.01) | .06 | 0.88 (0.76-1.00) | .03 |
| Communication concern | 0.9 (0.87-0.94) | <.001 | 0.96 (0.92-1.01) | .09 | 0.96 (0.92-1.01) | .14 |
| Social concern | 0.93 (0.87-0.98) | .007 | 0.97 (0.89-1.05) | .40 | 0.95 (0.87-1.03) | .23 |
| Any concern noted | 0.91 (0.88-0.93) | <.001 | 0.96 (0.93-1.00) | .06 | 0.96 (0.92-1.00) | .08 |

Results are adjusted for maternal age, maternal weight, SIMD decile, ethnicity, smoking history, illicit drug use, induction of labour, parity, previous CS, previous spontaneous or therapeutic abortion, pre-eclampsia, diabetes, gestational age, birthweight, year of birth, and sex of neonate.

© 2021 Kearns RJ et al. *JAMA Network Open.*

**eTable 4.** Estimation of Direct and Indirect Effects of Epidural Mediated by Mode of Delivery on Outcomes Referent to Receiving no Epidural (RR = 1)

| | Direct effect | | Indirect effect | | Total effect | |
|---|---|---|---|---|---|---|
| | **RR (95 % CI)** | ***P* value** | **RR (95 % CI)** | **P value** | **RR (95 % CI)** | **P value** |
| Neonatal resuscitation | 0.83 (0.79-0.86) | <.001 | 1.29 (1.27-1.31) | <.001 | 1.07 (1.03-1.11) | .003 |
| Apgar < 7 at 5 minutes | 0.74 (0.69-0.79) | <.001 | 1.25 (1.23-1.28) | <.001 | 0.92 (0.86-0.99) | .02 |
| Admitted to neonatal unit | 0.94 (0.91-0.97) | <.001 | 1.21 (1.20-1.22) | <.001 | 1.14 (1.11-1.17) | <.001 |
| Any concern noted | 0.95 (0.93-0.98) | <.001 | 1.00 (0.99-1.01) | .43 | 0.96 (0.93-0.98) | .001 |

Imputed data was used with results adjusted for maternal age, maternal weight, SIMD decile, ethnicity, smoking history, illicit drug use, induction of labour, parity, previous CS, previous spontaneous or therapeutic abortion, pre-eclampsia, diabetes, gestational age, birthweight, year of birth, and sex of neonate. The epidural was classed as the exposure of interest with the mode of delivery mediating the effect.

© 2021 Kearns RJ et al. *JAMA Network Open.*

**eTable 5.** Imputed Data: Characteristics of Women With and Without Educational Outcomes (After Exclusion of Data Missing for Anesthetic Type)

| **Total (n = 435,281)** | | **Missing data** | **Educational outcome (n = 220,508)** | **No educational outcome (n = 214,773)** |
|---|---|---|---|---|
| Age of mother | | 0 | 29 (24–33) | 29 (25–33) |
| Weight of mother | | 58,507 | 67 (59–79) | 67 (59–78) |
| Height of mother | | 59,489 | 164 (160–168) | 164 (160–168) |
| Ethnic group | Asian | 199,369 | 5,418 (4.0%) | 5,162 (5.2%) |
| | Black | | 1,795 (1.3%) | 1,985 (2.0%) |
| | Mixed | | 516 (0.4%) | 519 (0.5%) |
| | Other | | 874 (0.6%) | 1,251 (1.3%) |
| | White | | 127,751 (93.7%) | 90,641 (91.0%) |
| SIMD decile[a] | 1 | 1,056 | 32,996 (15.0%) | 27,044 (12.6%) |
| | 2 | | 28,244 (12.8%) | 24,924 (11.6%) |
| | 3 | | 24,998 (11.4%) | 23,233 (10.8%) |
| | 4 | | 23,167 (10.5%) | 21,856 (10.2%) |
| | 5 | | 21,629 (9.8%) | 20,920 (9.8%) |
| | 6 | | 20,402 (9.3%) | 19,503 (9.1%) |
| | 7 | | 19,408 (8.8%) | 19,552 (9.1%) |
| | 8 | | 18,447 (8.4%) | 19,880 (9.3%) |
| | 9 | | 16,422 (7.5%) | 19,172 (9.0%) |
| | 10 | | 14,360 (6.5%) | 18,068 (8.4%) |
| Smoker during pregnancy | Current | 27,529 | 45,036 (21.6%) | 35,748 (18.0%) |
| | Former | | 27,223 (13.0%) | 24,098 (12.1%) |
| | Never | | 136,546 (65.4%) | 139,101 (69.9%) |
| Injected illicit drugs – YES | | 154,775 | 1,730 (1.0%) | 677 (0.6%) |
| Spontaneous abortion – YES | | 1,180 | 50,465 (23.0%) | 47,074 (22.0%) |
| Therapeutic abortion – YES | | 1,177 | 18,681 (8.5%) | 16,996 (7.9%) |
| Parity | | 1,690 | 1 (0–1) | 1 (0–1) |
| Previous CS | | 1,519 | 0 (0–0) | 0 (0–0) |
| Induction | | 3,071 | 65,557 (30.0%) | 62,127 (29.1%) |
| Est gestation | | 0 | 40 (39–41) | 40 (39–41) |
| Birthweight | | 460 | 3,440 (3,100–3,780) | 3,460 (3,120–3,790) |
| Male sex | | 16 | 112,915 (51.2%) | 108,232 (50.4%) |
| Pre–eclampsia | | 0 | 2,679 (1.2%) | 2,641 (1.2%) |

Data are no. (%) or median (IQR). [a]The degree of social deprivation was categorised using deciles according to the Scottish Index of Multiple Deprivation (SIMD) with deciles of 1 (most deprived) to 10 (least deprived).

© 2021 Kearns RJ et al. *JAMA Network Open.*

**eTable 6.** Association of Epidural on Childhood Outcomes for Children Born Between January 1, 2012, December 31, 2016, Inclusive Referent to Receiving no Epidural (RR = 1)

| | Epidural (unadjusted) | | Epidural (confounder adjusted) [Cadj] | | Epidural (confounder and mediator [mode of delivery] adjusted) [CMadj] | |
|---|---|---|---|---|---|---|
| | **RR (95 % CI)** | ***P* value** | **RR (95 % CI)** | **P value** | **RR (95 % CI)** | **P value** |
| Offspring - gross motor concern at age 2-years | 0.87 (0.78-0.96) | .006 | 0.98 (0.88-1.10) | .76 | 0.97 (0.87-1.09) | .61 |
| Offspring - fine motor concern at age 2-years | 0.84 (0.76-0.93) | <.001 | 0.94 (0.85-1.04) | .24 | 0.94 (0.84-1.04) | .24 |
| Offspring -communication concern at age 2-years | 0.92 (0.88-0.95) | <.001 | 0.97 (0.93-1.01) | .11 | 0.97 (0.93-1.01) | .13 |
| Offspring - social concern at age 2-years | 0.92 (0.86-0.98) | .01 | 0.97 (0.91-1.04) | .46 | 0.96 (0.89-1.03) | .25 |
| Offspring - any concern at age 2-years | 0.92 (0.89-0.95) | <.001 | 0.96 (0.93-0.99) | .02 | 0.95 (0.92-0.99) | .009 |

Imputed data was used with results adjusted for maternal age, maternal weight, SIMD decile, ethnicity, smoking history, illicit drug use, induction of labour, parity, previous CS, previous spontaneous or therapeutic abortion, pre-eclampsia, diabetes, gestational age, birthweight, year of birth, and sex of neonate.

© 2021 Kearns RJ et al. *JAMA Network Open.*

**eFigure 1.** Love Plot Illustrating the Balance in Covariates Between Unmatched (red) and Matched (blue) Data Sets

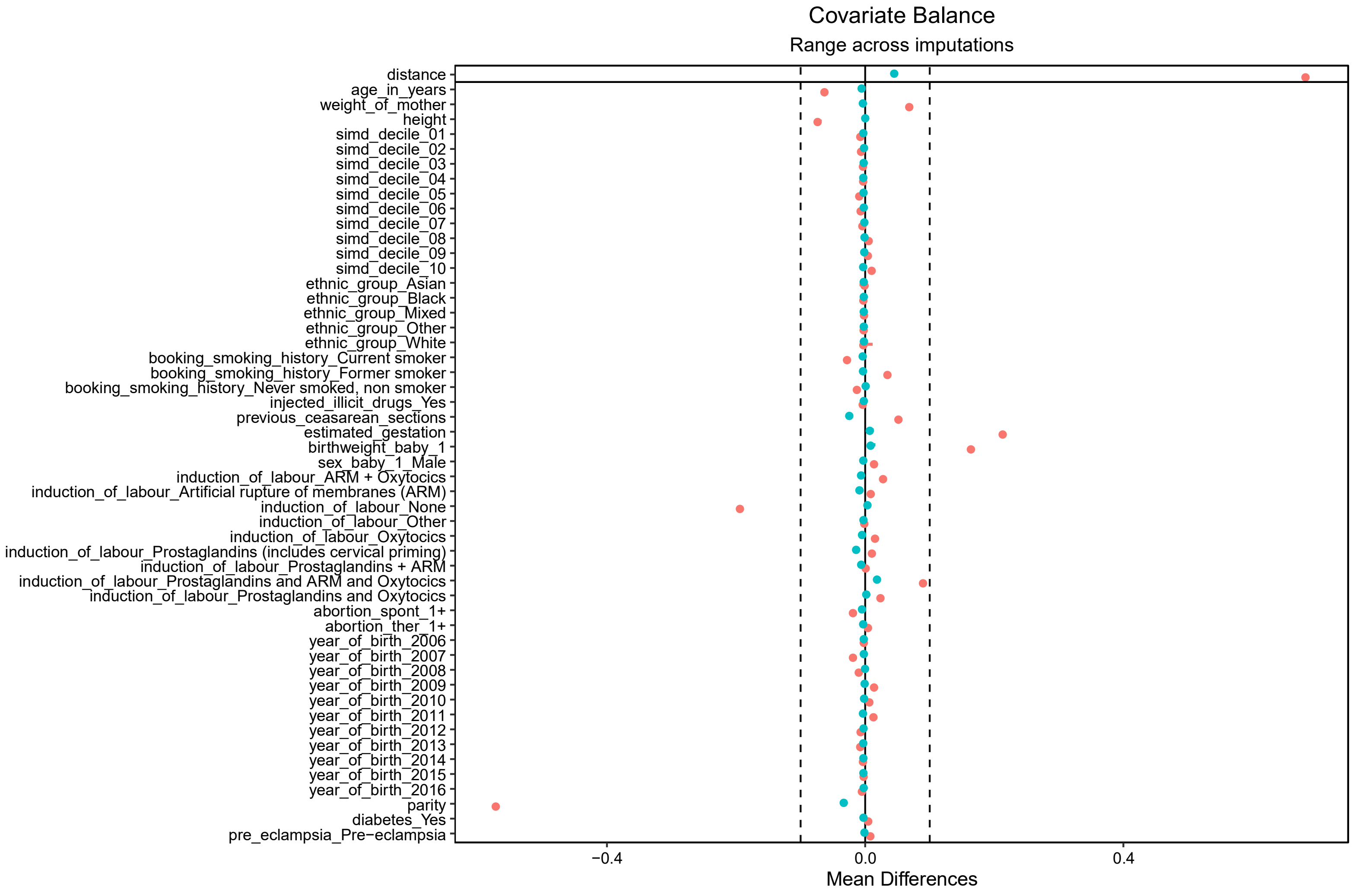


Standardized mean difference of 0.1 shown as dashed line.

© 2021 Kearns RJ et al. *JAMA Network Open.*

**eFigure 2.** Love Plot Illustrating the Balance in Covariates Between Unmatched (red) and Matched (blue) for the Health Care Use and Educational Outcomes Data Sets

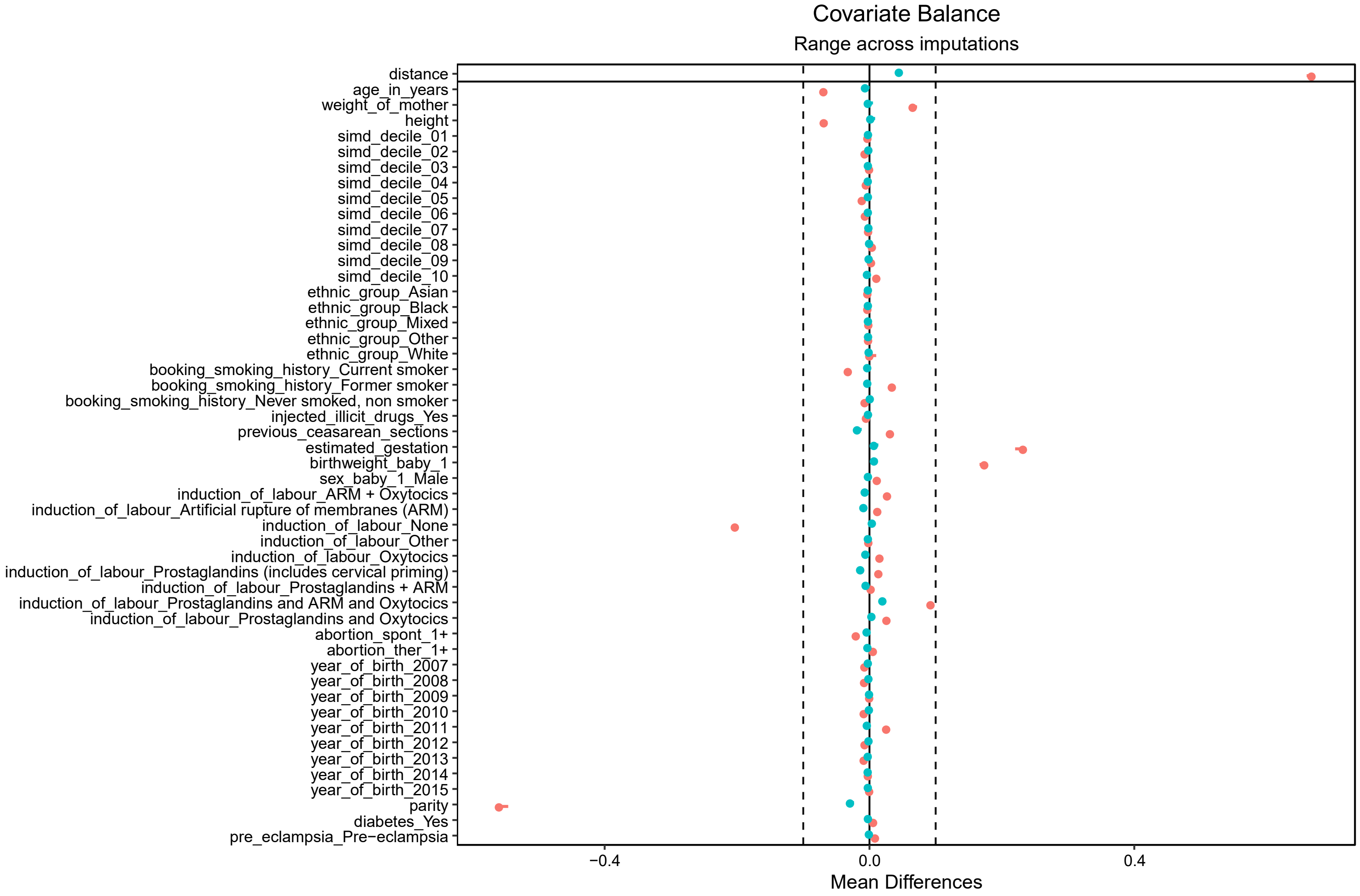


Standardized mean difference of 0.1 shown as dashed line.

© 2021 Kearns RJ et al. *JAMA Network Open.*

**eFigure 3.** Time-Varying Absolute Risks for Each Outcome in Relation to Gestational Age in Weeks

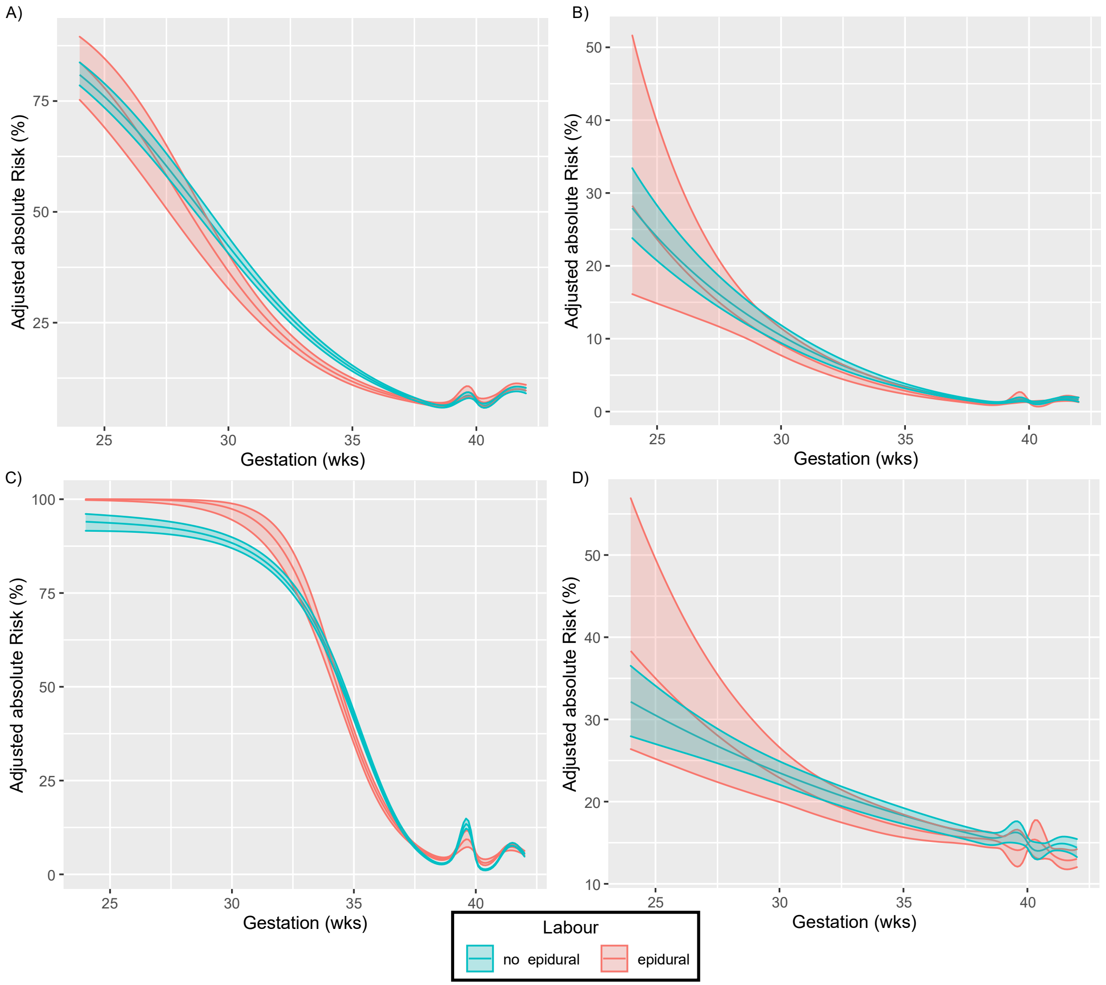


The expected value with 95% CI are shown for women with an epidural and no epidural for each gestational age week.
Panel A shows the Absolute Risk of Neonatal Resuscitation, Panel B Apgar <7 at 5 minutes, Panel C admitted to neonatal unit and panel D any developmental concern noted.

© 2021 Kearns RJ et al. *JAMA Network Open.*
